# Discovery of adipocyte-like accessory reproductive cells in male giant squid

**DOI:** 10.17912/micropub.biology.001494

**Published:** 2025-03-12

**Authors:** Noritaka Hirohashi, Yoshiaki Tanaka, Seiji Sasai, Tomohiro Sasanami, Hiroki Ono, Yoko Iwata, Eiji Fujiwara, Keisuke Yoshikiyo, Miwa Tamura-Nakano

**Affiliations:** 1 Department of Life Sciences, Faculty of Life and Environmental Science, Shimane University, Matsue, Shimane 690-8504, Japan; 2 Shimane Aquarium, Hamada, Shimane 697-0004, Japan; 3 Echizen-Matsushima Aquarium, Sakai, Fukui 913-0065, Japan; 4 Department of Applied Life Sciences, Faculty of Agriculture, Shizuoka University, Shizuoka, Shizuoka 422-8529, Japan; 5 Marine Biological Science Section, Education and Research Center for Biological Resources, Faculty of Life and Environmental Science, Shimane University, Okinoshima, Shimane 685-0024, Japan; 6 Atmosphere and Ocean Research Institute, The University of Tokyo, Chiba 277-8564, Japan; 7 Documentary Channel Co., Ltd., Tsurugashima, Saitama 350-2204; 8 Institute of Agricultural and Life Sciences, Academic Assembly, Shimane University, Matsue, Shimane 690-8504, Japan; 9 S-Nanotech Co-Creation Co., Ltd., Shimane University, Matsue, Shimane 690-8504, Japan; 10 Research Institute, National Center for Global Health and Medicine, Tokyo 162-8655, Japan

## Abstract

Some deep-sea squids are different from most other cephalopods, but similar to most animals, in their method of utilizing male genitalia. We conducted anatomical investigations of the male reproductive tract in the giant squid, *Architeuthis dux* and found that the lumens of the spermatophoric complex are filled with a myriad of lipid droplet-enriched cells. These cells have a spherical shape, consisting of a nucleus, lipid droplets making up approximately 25% of the cell, and well-developed rough endoplasmic reticulum. Chemical and proteomic analyses identified fatty acids and many abundant proteins that are common in their muscle tissues and mammalian adipocytes, respectively.

**Figure 1. Accessory reproductive cells in the giant squid spermatophoric complex.**
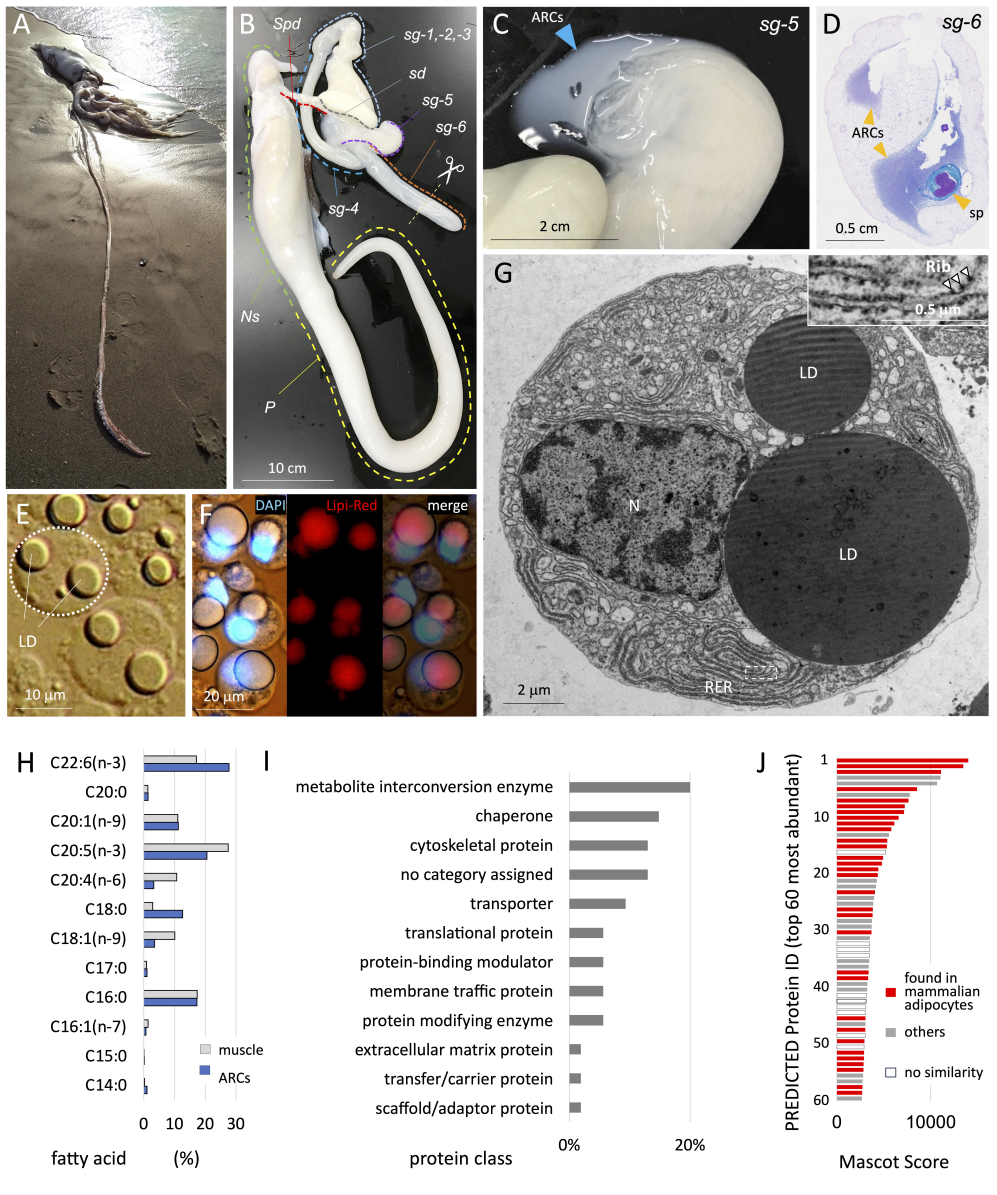
**A,** a male giant squid stranded on the coast of Torii, Oda, Japan on April 4, 2021. **B,** a whole view of a male reproductive organ (the spermatophoric complex) without testis. spermatophoric gland, sg-1 to sg-6; spermatophoric duct, Spd; Needham's sac, Ns; penis, P. **C,** luminal fluid exuded after cutting the epithelium of sg-5. **D,** a cross-section image at sg-6 (finishing) stained with PAS/Alcian Blue. ARCs, accessory reproductive cells; sp, spermatophore. **E,** a bright field image of accessory reproductive cells (ARCs) showing lipid droplets (LD). **F,** ARCs were stained with DAPI ( *left* ), Lipi-Red ( *middle* ) and their merged image ( *right* ). **G,** a transmission electron microscopic image of ARC showing nucleus (N), lipid droplets (LD), and rough endoplasmic reticulum (RER). Inset shows ribosomes (Rib). **H,** fatty acid compositions and their abundance (%) extracted from ARCs and female muscle tissues. **I,** a protein classification profile using proteomic and GO-based enrichment analyses (Carrera, 2019). **J,** shown are the Mascot scores of top 60 most abundant proteins that are present ( *red* ) or absent ( *gray* ) in mammalian adipocytes. Proteins with "no similarity" were shown in open columns.

## Description

Male internal fertilizers most commonly use their genitalia (or a penis) to deliver and deposit spermatozoa into the female reproductive tract. During this process, males often include other contents in amorphous ejaculates (or semen) that can facilitate fertilization success of their own sperm either directly such as nutrients or indirectly such as parasperm (Hayakawa, 2007; Toragall, 2019; Ramm, 2020). There is another type of internal fertilization where males transfer and deposit a spermatophore, which is a pre-packaged capsule enclosing concentrated spermatozoa, to the female. This type is known to occur in various taxa, such as annelids, arachnids, crustaceans, insects, and mollusks (Mann, 1984). Typically, the spermatophore is attached to the surface of the female's body, where sperm are stored for some time. In cephalopods, most species so far investigated are commonly using this "spermatophore" method (Marian, 2014). During copulation, the spermatophore undergoes morphological changes that involve shedding the outer tunic and exposing a sticky sperm-containing envelope called the spermatangium. The spermatangium then serves as a sperm reservoir for varying lengths of time, ranging from temporary (hours) to lifelong (months), depending on the species, site of deposition, and the male's mating tactics (Sato, 2021; Azad, 2024). Unlike other squids, females of some deep-sea squids have been observed to have the spermatangium embedded under their skin in random places (Nesis, 1995; Hoving, et al., 2010b; Murai, 2021). In parallel with this, males of these species possess a relatively longer terminal organ that is morphologically and functionally analogous to a penis, rather than a distinctive hectocotylus (but see Nesis, 1995; Hoving et al., 2004). The hectocotylus is one of the arms of male cephalopods that is specialized for storing and transferring the spermatophore to females. The current leading hypothesis on the mating behavior of deep-ocean squids suggests that they use the terminal organ to transfer the spermatophore directly on/under the female's skin. In agreement with this, an underwater expedition using a remotely operated vehicle captured the moment of mating behavior of *Pholidoteuthis adami* using the terminal organ passing through the funnel, which is a ventrally located, cylinder-shaped water jetting apparatus (Hoving, 2012). More recently, a male giant squid, *A. dux* ( Fig. 1A ) *,* was found in shallow water exhibiting the behavior of "spermatophore expelling" with its terminal organ passing through the funnel (Sasai et al., 2025 *submitted* ), suggesting that direct spermatophoric transfer also occurs in *A. dux.* However, the evolutionary transition from hectocotylus to terminal organ as a mating style remains an enigmatic issue.

We carried out anatomical investigations of male reproductive tract (the spermatophoric complex, Fig. 1B ) in *A. dux* . We found large spherical cells with a diameter of approximately 20 mm (Figs. 1C-1E) to be abundant in the lumens of most parts of the spermatophoric complex, including the spermatophoric gland ( *Sg-4, -5, -6* ), spermatophoric duct ( *Spd* ), Needham's sac ( *Ns* ) and a penis ( *P* ) as shown in Fig. 1B . Here, we have coined these cells as Accessory Reproductive Cells (ARCs). ARCs contained oil droplets (Figs. 1E-1G). The transmission electron microscopy revealed a highly conserved intracellular structure with relatively simple organelle compositions, such as a nucleus, lipid droplets and rough endoplasmic reticulum (RER). Since lipid droplets make up approximately 1/4 of the cell volume (25.1 ± 6.3%, n=9), we speculate that ARCs may play a role in lipid storage. Next, we compared the fatty acid compositions in ARCs with those in the muscle tissue of the same female. There were no unique fatty acid species in ARCs; instead, their profiles were more or less similar to those found in muscles ( Fig. 1H ). Finally, we took a proteomic approach to gain insight into the function of ARCs. A total of 1,906 proteins were successfully predicted to be present in the ARCs ( Fig. 1I, Extended Data file on figshare https://doi.org/10.6084/m9.figshare.28106831.v1), of which 51.7% of the top 60 most abundant proteins were also found in mammalian adipocytes ( Fig. 1J, Extended Data file). These results suggest that ARCs may have a primarily role in energy storage.

Lipid droplets are common intracellular organelles found in most cells (Zadoorian, 2023). Although their functions are versatile, their major roles are to store and supply cellular energy. We found that the lumens of the spermatophoric complex are filled with ARCs that can be released from the tip of the terminal organ. If male giant squids were to pursue direct copulation with females using their terminal organ as is assumed in some deep-sea squid species, ARCs would be transported to the female tissues along with the spermatangium. Thereafter, lipids in ARCs may be utilized to fuel energy to sperm cells at the site of sperm storage within the female. In species with long-term sperm storage in females, sperm often contain lipid droplets that could act as a source of endogenous energy (Zhang et al., 2015). Unlike other marine invertebrates, the sperm of octopuses and squids are known to uptake extracellular glucose and fructose to generate ATP through intrinsic glycolytic pathways (Austin et al., 1964; Hirohashi et al., 2016). Similarly, *A. dux* sperm may utilize lipid metabolites either directly or indirectly through gluconeogenesis. Due to their large body size, the distances between the sites of sperm storage and the site of egg release are quite far (Murai et al., 2021), so additional extracellular energy supply may be necessary to help the sperm travel and reach the eggs. Alternatively, ARC itself or released lipids may function as a lubricant in the male terminal organ, facilitating the smooth and quick transport of the spermatophores to the opening end of the terminal organ.

## Methods

**Collection of specimens**

In this study, we obtained two male specimens that had stranded as either dead or dying animals on the coasts of the Sea of Japan. The first dead specimen was collected at Torii-kaigan, Oda-shi, Shimane, Japan on April 4, 2021 ( Fig. 1A ). The specimen was a mature male with a mantle length of 1,246 mm and a total length of 5,740 mm. The second specimen was found in shallow water in a deadly condition as previously reported (Sasai et al., 2025 *submitted* ). Soon after dying, the specimen was retrieved from the water and transported to the Echizen-Matsushima Aquarium.

**Transmission electron microscopy**

Samples were pre-fixed with 2.5% glutaraldehyde in 30 mM HEPES buffer (pH 7.4) containing 100 mM NaCl, 2 mM CaCl _2_ and 0.45 M sucrose for 24 h at 4°C, and postfixed with 1% osmium tetroxide and 10 mM potassium ferricyanide in 30 mM HEPES buffer (pH 7.4) containing 100 mM NaCl, 2 mM CaCl _2_ for 1.5 h at room temperature. The following steps including dehydration, embedding, ultra-thin sectioning, electron staining, and imaging were carried out as previously described (Hirohashi et al., 2016).

**Histology**

Paraffin sections were stained with PAS/Alcian Blue (pH2.5) according to the standard procedure. The nucleus and lipid droplets in ARCs were stained with 1mg/ml 4',6-diamidino-2-phenylindole (DAPI, Thermo Fisher Scientific) and 1mg/ml Lipi-Red (Dojindo Co., Ltd.), respectively and visualized under epifluorescence microscopy (Nikon TE-2000).&nbsp;&nbsp; 

**Fatty acid analysis by GC-MS**

Fatty acids were analyzed using a Shimadzu GCMS-QP2010 Ultra spectrometer equipped with a quadrupole mass detector (1.00 ± 0.10 kV) and a DB-5ms 30 m × 0.25 mm i.d. capillary column with a film thickness of 0.25 µm (Agilent Technologies, Santa Clara, CA, USA) as described previously (Yoshikiyo et al., 2023). Free and esterified fatty acids in muscle or ARCs were converted to their methyl esters using reagent kits following manufacturer protocols (Nacalai Tesque Inc., Kyoto, Japan).

**Proteomic analysis**

A suspension of ARCs (25 ml) was diluted into 200 ml denaturing buffer (9 M urea, 50 mM Tris-HCl pH 8.0), sonicated and its protein concentration was measured using a BCA assay kit. The sonicated sample (10 mg of total protein) was then subjected to 12.5% SDS-PAGE, followed by CBB Stain (Nakalai tesque). In-gel digestion with trypsin was performed according to (Shevchenko, et al., 1996). Dehydrated peptide samples were dissolved in solvent (H2O/acetonitrile/trifluoroacetic acid = 98/2/0.1), subjected to LC-MS/MS (Ultimate 3000 RSLCnano with a Nano HPLC Capillary Column, Q Exactive Orbitrap, Thermo Fisher Scientific). The peaks were analyzed using Proteome Discoverer (ver. 2.2) (https://www.thermofisher.com/jp/ja/home.html) and MASCOT Peptide Mass Fingerprint to identify proteins from a nonhuman proteome database (The Global Proteome Machine Organization: http://www.thegpm.org/crap/index.html). A total of 1,906 proteins were identified (figshare https://doi.org/10.6084/m9.figshare.28106831.v1).

Data Availability: Proteomic data are available on figshare at https://doi.org/10.6084/m9.figshare.28106831.v1
